# Association between SNPs of Circulating Vascular Endothelial Growth Factor Levels, Hypercholesterolemia and Metabolic Syndrome

**DOI:** 10.3390/medicina55080464

**Published:** 2019-08-11

**Authors:** Ali Salami, Said El Shamieh

**Affiliations:** 1 Rammal Hassan Rammal Research Laboratory, Physio-toxicity (PhyTox) Research Group, Faculty of Sciences (V), Lebanese University, Nabatieh 1700, Lebanon; 2 Department of Medical Laboratory Technology, Faculty of Health Sciences, Beirut Arab University, Beirut 115020, Lebanon

**Keywords:** single nucleotide polymorphisms, VEGF, hypercholesterolemia, metabolic syndrome

## Abstract

*Background and Objectives*: Four single nucleotide polymorphisms (SNPs); rs6921438 and rs4416670 in *LOC100132354*-*C6orf223*, rs6993770 in *ZFPM2,* and rs10738760 in *VLDLR*-*KCNV2* were reported to explain up to 50% of the heritability of vascular endothelial growth factor circulating levels. These SNPs were also studied for possible associations with circulating lipid levels in supposedly healthy European individuals and in a limited number of Iranian individuals with metabolic syndrome. To go further, the association of those four SNPs with plasma lipid parameters, hypercholesterolemia and metabolic syndrome (MetS) was assessed. *Materials and Methods*: A cross-sectional study was conducted on 460 individuals chosen from the general population. Demographic and clinical data were collected and DNA was extracted and genotyped using Kompetitive allele specific PCR (KASP™). A meta-analysis followed, combining our participants with the Iranian individuals (*n* = 336). *Results*: Whereas rs10738760 was associated with total cholesterol (Tchol) (*p* = 0.01), rs6993770 showed significant associations with both Tchol and low-density lipoprotein cholesterol (LDL-C) levels (*p* = 0.007 and *p* = 0.01 respectively). Using a multivariate logistic regression model adjusted for different confounding factors, we found that rs6993770 was associated with hypercholesterolemia, specifically high Tchol (*p* = 0.01) and LDL-C levels (*p* = 0.01). Furthermore, rs10738760 was positively associated with the risk of MetS in these individuals (*p* = 0.02) and in the meta-analysis (OR = 1.67, *p* = 0.01). *Conclusion*: Our results suggest that whereas rs6993770 in *ZFPM2* was positively associated with hypercholesterolemia, rs10738760 (*VLDLR*-*KCNV2*) has a possible implication in MetS in two Middle Eastern populations.

## 1. Introduction

Blood lipids are consistently associated with cardiovascular disease (CVD) events, in particular, such events are associated with high levels of low-density lipoprotein cholesterol (LDL-C) [1]. Unlike LDL-C, high-density lipoprotein cholesterol (HDL-C) has received much attention in the past few years as a result of increased knowledge of its atheroprotective effects [2]. 

The vascular endothelial growth factor (VEGF, also referred to as VEGFA) family is an important regulator of vascular biology [3]. Specifically, it stimulates angiogenesis in a wide range of processes (both normal and pathological) [3]. Due to its significant role in blood vessel homeostasis, the contribution of VEGF to CVD and atherogenesis has been reported in recent years [4]. It was reported that VEGF levels may mediate inflammation and neovascularization in atheromatous plaques in mice [5]. In humans, increased plasma VEGF levels have been found in patients with coronary heart disease [6] and with hyperlipidemia [7]. Furthermore, measured VEGF levels were associated with traditional CVD risk factors such as lipid profile, blood pressure, and body mass index [8]. 

The heritability of circulating VEGF levels is high and estimated to be between 60% and 80% [9]. In a genome-wide association study (GWAS), Debette et al. identified four single nucleotide polymorphisms (SNPs); rs6921438 and rs4416670 in *LOC100132354*-*C6orf223*, rs6993770 in *ZFPM2,* and rs10738760 in *VLDLR*-*KCNV2,* that explained up to 50% of the heritability of VEGFA circulating levels [10]. These SNPs were further studied for possible relationships with lipid parameters and this revealed that rs6921438 was associated with decreased HDL-C and increased LDL-C levels in supposedly healthy European individuals [11]. Recently, rs6993770 was reported by Azimi et al. to be associated with metabolic syndrome (MetS) in a relatively limited Iranian population [12].

To go further, this study investigated the association of those four SNPs with plasma lipid parameters and hypercholesterolemia in a Lebanese general population (*n* = 460). To confirm the relation with MetS, the relationship with MetS in this population was first tested, and then a meta-analysis was conducted combining these participants with Iranian participants (*n* = 336) from the study of Azimi et al. [12].

## 2. Materials and Methods

### 2.1. Ethics Statement

Participants involved in the present study were recruited in accordance with the latest version of the Declaration of Helsinki for Ethical Principles for Medical Research Involving Human Subjects as described previously [13]. Protocols for the genetic studies were approved by the local ethics committees for the protection of subjects for biomedical research. This study was approved by the institutional review board of the Lebanese University (Approval reference number: 2182/28, date: 16/12/2015).

### 2.2. Study Population

The participants were selected from a cross-sectional population-based study involving Lebanese unrelated individuals free of chronic disease (cardiovascular or cancer) with recruitment taking place from 2015 to 2016 in a major tertiary care hospital.

### 2.3. Clinical and Biological Data Collection

Demographic and clinical measurements (weight, height, and blood pressure) were assessed. Blood samples were collected according to the manufacturer’s recommendations for assays. Biochemical measurements including lipid parameters were acquired with commercial kits as described previously [14]. Hypercholesterolemia was defined as total cholesterol levels ≥200 mg/dL or LDL-C levels ≥100 mg/dL, obesity as body mass index (BMI) ≥30 kg/m^2^. Hypertension (HTN) was defined as systolic blood pressure ≥130 mmHg or diastolic blood pressure ≥85 mmHg. MetS was defined according to the International Diabetes Federation (IDF) criteria.

A systematic DNA collection was implemented for every participant according to international ethical regulations and good laboratory practices. Total genomic DNA was extracted from peripheral blood samples according to manufacturer recommendations (QIAamp DNA blood mini kit, Qiagen, Hilden, Germany). Genotyping was performed by the LGC group (Berlin, Germany) using a KASP genotyping assay as described previously [15].

### 2.4. Statistical Analysis

All analyses were performed using SPSS statistical software version 24.0 (SPSS, Inc.; Chicago, IL, USA). Continuous variables are presented as mean value ± standard deviation, and categorical variables are given as number and percentages. To determine if the genotypes were in Hardy–Weinberg equilibrium, a chi-square test was performed. All genetic analyses were performed under the assumption of an additive model.

Linear regression models adjusted for age, gender, body mass index (BMI), marital status, income, smoking, and physical activity were used for the assessment of the effect of each SNP on blood lipid levels. The significance level was set at *p* ≤ 0.01 due to multiple testing. The contribution of “SNP x gender” interaction was also assessed within our model; the significance level was set at *p* ≤ 0.05.

To study the association between the SNPs and dyslipidemia, a multivariate logistic regression model was used, while correcting for all potential confounding factors including age, gender, BMI, marital status, smoking, alcohol consumption, and physical activity. The significance level was set at *p* ≤ 0.01 due to multiple testing. A similar model was used to assess the effect on MetS with a significance level of *p* ≤ 0.025.

### 2.5. Meta-Analysis with Metabolic Syndrome

Meta-analysis was conducted using Comprehensive Meta-Analysis software “V3”; calculations were done using a random effects method. Statistical heterogeneity of the treatment effects among studies was assessed using Cochran’s Q and the inconsistency *I^2^* tests, in which values above 25% and 50% were considered indicative of moderate and high heterogeneity, respectively [16]. The level of significance was set at *p* ≤ 0.05.

## 3. Results

Table 1 shows the demographic characteristics of the participants. 

In addition, the clinical, biochemical, and genetic characteristics were shown in Table 2. According to international classification criteria, approximately 75% of our participants had high total cholesterol and LDL-C levels; approximately half had low HDL-C. 

All tested SNPs were in agreement with the Hardy–Weinberg equilibrium. Associations among genetic variants and blood lipid concentration traits are shown in Table 3. Whereas rs10738760 was associated with total cholesterol (Tchol) (*p* = 0.01, Table 3), rs6993770 showed significant associations with both Tchol and LDL-C levels (*p* = 0.007 and *p* = 0.01, respectively, Table 3). Similarly, gender also showed a significant association with Tchol, HDL-C, and LDL-C levels (*p* < 0.001, respectively, Table 3). An interaction between rs6993770 and gender, positively influencing Tchol and LDL-C levels, was also observed (*p* < 0.05, Table 3). No significant associations were found with HDL-C and triglycerides (data not shown). 

Next, a multivariate logistic regression model adjusted for different confounding factors was applied leading to the finding that rs6993770 was associated with hypercholesterolemia, specifically high Tchol (*p* = 0.01, Table 4) and LDL-C levels (*p* = 0.01, Table 4). Among other dependent variables, age, BMI, and smoking were found to increase the risk of having high Tchol levels (*p* ≤ 0.03). Being married and doing physical activity decreased the risk of high Tchol and LDL-C remarkably (Table 4). In contrast to rs6993770, rs10738760 was positively associated with the risk of MetS (*p* = 0.02, Table 4).

**Figure 1:**
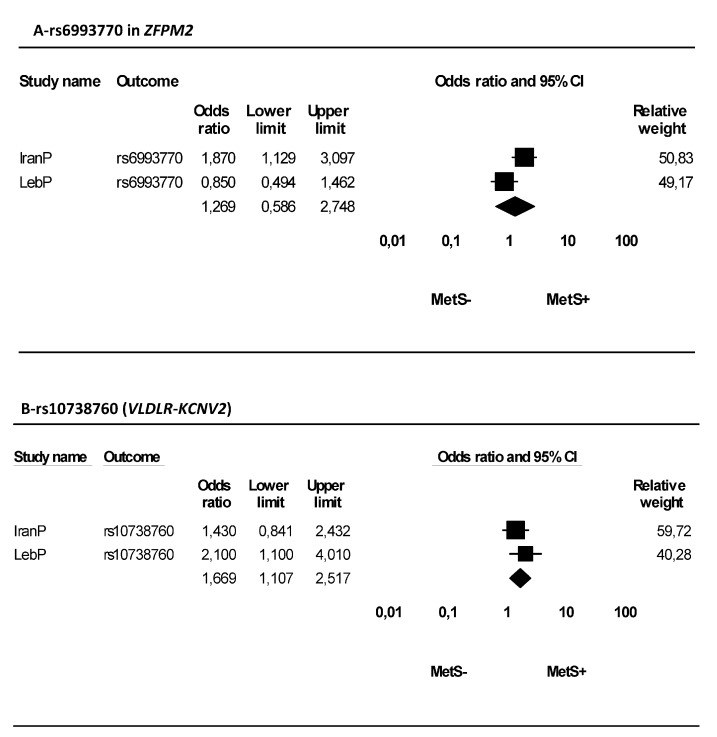
Forest plot for meta-analysis of rs6993770 in *ZFPM2* and rs10738760 (*VLDLR*-*KCNV2*). (**A**) Heterogeneity: Tau^2^ = 0.24, Chi^2^ = 4.35, df = 1 (*p* = 0.04); *I*^2^ = 77.02%. Test for overall effect: Z = 0.60 (*p* = 0.55). (**B**) Heterogeneity: Tau^2^ = 0.00, Chi^2^ = 0.81, df = 1 (*p* = 0.37); *I*^2^ = 0.00%. Test for overall effect: Z = 2.45 (*p* = 0.01).

A forest plot for meta-analysis of rs6993770 and rs10738760 is shown in Figure 1. No association between rs6993770 and MetS was found, and a significant heterogeneity between this study and the study conducted by Azimi et al. [12] was also seen (*p* = 0.04 and *I*^2^ = 77.02%, Figure 1A). In contrast, rs10738760 significantly increased (OR = 1.67, *p* = 0.01) the risk of MetS (Figure 1B), chi-square and I^2^ tests showed no heterogeneity (*p* = 0.37 and *I*^2^ = 0%, respectively, Figure 1B).

## 4. Discussion

The results indicate that whereas rs10738760 was associated with Tchol, rs6993770 showed significant associations with both Tchol and LDL-C levels. An interaction between rs6993770 and gender, positively influencing Tchol and LDL-C levels, was also observed. Using a multiple logistic model, the results demonstrate that rs6993770 was associated with increased risk of hypercholesterolemia through its positive association with Tchol and LDL-C. Furthermore, rs10738760 was positively associated with higher risk of MetS in our population and in the meta-analysis with Azimi et al.’s participants [12].

Population based studies have demonstrated differences in prevalence by gender and ethnicity for hypercholesterolemia and MetS [17,18]. A recent survey conducted by National Center for Health Statistics reported that during 2015–2016, 12.4% of adults had high Tchol and 18.4% had low HDL-C [17]. Although there were no significant race and Hispanic-origin differences in high total cholesterol overall or in men, non-Hispanic white women had a higher prevalence of high Tchol than Hispanic women [17]. The prevalence of low HDL-C was lowest in non-Hispanic black adults and highest in Hispanic adults overall and for men [17]. On the other hand, the prevalence of MetS among Lebanese adults aged 18–65 years old was shown to be ~31% with men presenting with a higher rate than women [19]. Going in the same direction, findings from the National Health and Nutrition Examination Survey reported that the prevalence of MetS among the US adult population was 34.0% [18]. While the prevalence of MetS was comparable between the two genders (men (35%) and women (33%)) in the US, it was higher among men in Lebanon. In contrast, if compared with surrounding countries such as Turkey [20], Oman [21], and Tehran [22], one could notice a higher incidence in women, possibly caused by higher prevalence of abdominal obesity in women than in men [20,21,22].

This is not the first study to show an association between *VEGF*-related polymorphisms; in a large two-stage population study of 2151 European individuals, Stathopoulou found that rs6921438 alone, and through interaction with rs6993770, was associated with HDL-C [11]. Combined with these findings, these observations point to a possible role of VEGF in atherosclerosis. This is supported by evidence in four different mouse models that demonstrated that VEGF-A induced changes in lipoprotein profiles and increased the triglyceride proportion of large VLDL particles [23].

Supporting the findings of this study, Azimi-Nezhad et al. reported an association between the rs6993770T allele and the presence of MetS, and this association remained significant after adjustment for confounding factors [12]. The meta-analysis combining the participants of this study with Iranians showed an overall significant effect for rs10738760 with regard to MetS and highlighted a possible implication for this SNP.

A negative association between alcohol consumption and hypercholesterolemia was found in the current study, this is unusual since low LDL-C in chronic alcoholics is well known [24]. Population-based studies showed that the effect of alcohol on many diseases has a biphasic pattern, depending on the amount of alcohol consumed [25,26], race/ethnicity, or concurrent disease state of participants in each study [27]. It is important to note that the association between rs6993770 and rs10738760 with hypercholesterolemia and MetS, respectively, has previously explained a minor fraction of VEGF plasma levels (2% and 5%, respectively), implying that VEGF might be a player in hypercholesterolemia and MetS, yet other additional molecules are still to be identified. Since VEGF levels were not quantified in the current study, we can never confirm this hypothesis. 

These findings are strengthened by the fact that the regression models used in this study were adjusted for several confounding covariates such as age, gender, marital status, smoking and physical activity. This study has two main limitations; (1) female participants were more prevalent than males with a ratio of 2:1, requiring adjustment for gender to remove any confounding effect and (2) the lack of replication in larger populations.

## 5. Conclusions

In conclusion, these results suggest that whereas rs6993770 in *ZFPM2* was positively associated with hypercholesterolemia, rs10738760 (*VLDLR*-*KCNV2*) is possibly implicated in MetS in two Middle Eastern populations.

## Figures and Tables

**Table 1:** Demographic characteristics of the study participants.

Characteristics	Participants (*n* = 460)
Age	40.60 ± 14.16
Gender *n* (%)	
Male	168 (36.5)
Female	292 (63.5)
Education Level *n* (%)	
None	3 (0.7)
School	194 (42.2)
University	263 (57.2)
Marital Status *n* (%)	
Single	121 (26.3)
Married	321 (69.8)
Divorced	18 (3.9)

Values are arithmetic mean ± SD for continuous variables. Categorical variables were shown as number (*n*) and percentages (%). *n*: Sample size.

**Table 2:** Clinical, biochemical, and genetic characteristics of the study participants.

Characteristics	Participants (*n* = 460)
BMI (Kg/m^2^)	25.71 ± 4.98
Alcohol consumption (current drinker) *n* (%)	162 (35.3)
Smoker *n* (%)	122 (26.5)
Physical activity (more than once per week) *n* (%)	115 (25.0)
Total cholesterol (mg/dL)	181.41 ± 40.94
High *n* (%)	351 (76.3)
LDL-cholesterol (mg/dL)	117.39 ± 33.52
High *n* (%)	347 (75.4)
HDL-cholesterol (mg/dL)	45.53 ± 14.61
Low *n* (%)	270 (58.7)
Triglycerides (mg/dL)	145.96 ± 124.34
High *n* (%)	174 (37.8)
SBP (mmHg)	132.07 ± 15.89
DBP (mmHg)	67.82 ± 9.12
Hypertension *n* (%)	255 (55.4)
MAF	
rs6921438G > A	0.34
rs4416670C > T	0.49
rs6993770A > T	0.34
rs10738760A > G	0.46

Values are arithmetic mean ± SD for continuous variables. Categorical variables were shown as number (*n*) and percentages (%). *n*: Sample size, MAF: Minor allele frequency.

**Table 3:** Multivariate linear regression analysis with lipoprotein profile.

Variables	SNP	Ancestral Alleles	MAF	*p*	Beta	SE	Trait
Age				<0.001	0.68	0.15	Tchol
				<0.001	2.07	0.45	HDL-C
				<0.001	0.53	0.13	LDL-C
Gender				<0.001	14.10	4.49	Tchol
				<0.001	5.93	1.56	HDL-C
				<0.001	11.15	3.75	LDL-C
*VLDLR-KCNV2*	rs10738760	A	0.46	0.01	–5.53	2.62	Tchol
*ZFPM2*	rs6993770	A	0.34	0.007	7.89	2.90	Tchol
				0.01	6.20	2.42	LDL-C
*ZFPM2*	rs6993770 x Gender	A		0.02	3.661	1.58	Tchol
				0.03	2.795	1.31	LDL-C

SNP: Single nucleotide polymorphism, MAF: Minor allele frequency, beta: Regression coefficient corresponding to the minor allele of each SNP, SE: Standard error, Tchol: Total cholesterol, HDL-C: High-density lipoproteins cholesterol, LDL-C: Low-density lipoproteins cholesterol. Regression analyses used to test SNPs–triglycerides associations were not shown since they were not significant.

**Table 4:** Multiple logistic regression analysis of risk factors with hypercholesterolemia and metabolic syndrome.

Risk Factors	Total Cholesterol		LDL-C		MetS	
	OR (95% C.I.)	*p*	OR (95% C.I.)	*p*	OR (95% C.I.)	*p*
Age						
<40	1		1		1	
≥40	22.67 (9.70–53.02)	<0.001	-	ns	-	ns
Gender						
Male	1		1		1	
Female	-	ns	-	ns	-	ns
BMI						
<25	1		1		1	
25–29.9	-	ns	-	ns	2.44 (1.29–4.62)	0.006
≥30	3.03 (1.16–7.92)	0.02	2.78 (1.06–7.33)	0.04	-	ns
Marital status						
Non-married	1		1		1	
Married	0.16 (0.07–0.35)	<0.001	-	ns	0.54 (0.28–1.03)	0.06
Divorced	-	ns	-	ns	-	ns
Widowed	-	ns	-	ns	-	ns
Smoking status						
Non smoker	1		1		1	
Past smoker	-	ns	-	ns	-	ns
Current smoker	2.24 (1.08–4.64)	0.03	-	ns	-	ns
Alcohol consumption						
Non drinker	1		1		1	
Past drinker	0.01 (0–0.07)	<0.001	0.01 (0.001–0.11)	<0.001	-	ns
Current drinker	0.47 (0.25–0.88)	0.01	-	ns	-	ns
Physical activity					1	
<1 per week	1		1			
1 per week	0.291 (0.12–0.68)	0.004	0.24 (0.10–0.58)	0.001	-	ns
≥2 per week	-	ns	-	ns	-	ns
rs6993770 AA	1		1		1	
AT	-	ns	-	ns	-	ns
TT	2.61 (0.94–7.19)	0.01	4.30 (1.37–13.51)	0.01	-	ns
rs10738760 AA	1		1		1	
GA	-	ns	-	ns	2.10 (1.10–4.01)	0.02
GG	-	ns	-	ns	-	ns

LDL-C: Low-density lipoprotein cholesterol, MetS: Metabolic Syndrome, ns: Not significant.
